# *Anisakis* larvae (Nematoda: Anisakidae): retrospective morphological, morphometric, biogeography, and taxonomic status analysis

**DOI:** 10.1590/S1984-29612025047

**Published:** 2025-10-17

**Authors:** Raul Henrique da Silva Pinheiro, Ricardo Luis Sousa Santana, Tallytha de Nazaré Paixão da Silva, Yan Rafael Gillet Santa Brigida, Luis Augusto Araújo dos Santos Ruffeil, Elane Guerreiro Giese

**Affiliations:** 1 Universidade Federal Rural da Amazônia – UFRA, Instituto da Saúde e Produção Animal – ISPA, Laboratório de Histologia e Embriologia Animal – LHEA, Belém, PA, Brasil

**Keywords:** Fish food safety, nematodes, zoonosis, Amazon, Segurança alimentar do pescado, nematódeos, zoonose, Amazônia

## Abstract

The family Anisakidae includes parasite genera that are important for public health due to their zoonotic potential. Among these, the genus *Anisakis* contains some of the most prevalent parasites found in fish that are consumed and commercially exploited in Brazil. Thus, this study aimed to investigate records of the presence of third-stage larvae of *Anisakis* spp. fish parasites found in Brazilian territory, focusing on their morphological, morphometric, biogeographic, and prevalence aspects over a period of 40 years. This analysis identified the presence of *Anisakis* larvae in 18 orders, 2 groups, 40 families, 60 genera and 69 species of infected marine, brackish and freshwater fish, demonstrating the lack of specificity to a particular group, which was also evident in the different morphometric data, as well as in the site of infection and habitat of the hosts, with predominantly marine fish being the most infected.. The presence of different *Anisakis* morphotypes highlights parasitic biodiversity and reinforces the need for taxonomic studies of these zoonotic agents found in fish consumed as food. Special attention should be given to the Amazonian ichthyofauna, located in one of the aquatic ecoregions considered a research priority in Brazil, with the identification of these zoonotic parasites being a matter of food security and public health.

## Resumo

A família Anisakidae agrupa gêneros de parasitos de alta importância para Saúde Pública, por apresentarem espécies com potencial zoonótico. O gênero *Anisakis* está entre os parasitos mais prevalentes em peixes consumidos e explorados comercialmente no Brasil. Assim, este estudo teve como objetivo investigar os registros parasitários da presença de larvas de terceiro estágio de *Anisakis* spp., parasito de peixes no território nacional, contemplando aspectos morfológicos, morfométricos, biogeográficos e de prevalência em um período de 40 anos. Esta análise identificou a presença de larvas de *Anisakis* em 18 ordens, 2 grupos, 40 famílias, 60 gêneros e 69 espécies de peixes marinhos, de águas salobras e dulcícolas infectados, demonstrando a não especificidade a um determinado grupo, o que também ficou evidenciado nos diferentes dados morfométricos, bem como no sítio de infecção e habitat dos hospedeiros, sendo os peixes predominantemente marinhos os mais infectados. A presença de diferentes morfotipos de *Anisakis* demonstra a biodiversidade parasitária e reforça a necessidade de estudos taxonômicos desses agentes zoonóticos presentes em peixes utilizados como alimento, com destaque para a ictiofauna amazônica, como umas das ecorregiões aquáticas consideradas prioritárias para a pesquisa no Brasil, sendo a identificação desses parasitos zoonóticos uma questão de segurança alimentar e saúde pública.

## Introduction

The nematode superfamily Ascaridoidea contains 52 genera, with parasitic species that affect the alimentary tract of vertebrates (Mattiucci & Nascetti, 2008). The family Anisakidae Railliet & Henry 1912 includes nematodes that are highly significant in both the medical and veterinary fields. These nematodes are known to cause emerging zoonotic diseases, specifically anisakidosis (Chai et al., 2005; Shamsi & Suthar, 2016). In Brazil, this is one of the most representative families in terms of the number of genera that may be associated with commercially important fish, with the following genera being recorded: *Anisakis* Dujardin, 1845; *Contracaecum* Railliet & Henry, 1912; *Peritrachelius* (Diesing, 1851); *Pulchrascaris* Vicente & Santos, 1972; *Pseudoterranova* Mosgovoi, 1951; *Skrjabinisakis* (Mozgovoy, 1951); and *Terranova* Leiper & Atkinson, 1914 (Luque et al., 2011; Safonova et al., 2021). And despite the biodiversity of genera, the species exhibit complex phylogenetic relationships, and many have been synonymized or have become *taxa inquirendum* (Nemys, 2024).

The classification and nomenclature of *Anisakis* (Nematoda: Anisakidae) were controversial and confusing until genetic and molecular methodologies began being applied. Over the last 20 years these methods have led to a stable and widely accepted taxonomy (Mattiucci & Nascetti, 2008). *Anisakis* consists of parasites with global distribution and complex life cycles that have capacity to potentially significantly influence aquatic ecosystems worldwide (Shamsi, 2021).

Worldwide, according to Nemys (2024), *Anisakis* is composed of 8 morphospecies, namely: *Anisakis simplex* (Rudolphi, 1809); *Anisakis similis* (Baird, 1853) Baylis, 1920; *Anisakis typica* (Diesing, 1860) Baylis, 1920; *Anisakis oceanica* (Johnston & Mawson, 1951) Davey, 1971; *Anisakis pegreffii* Campana-Rouget & Biocca, 1955; *Anisakis ziphidarum* Paggi, Nascetti, Webb, Mattiucci, Cianchi & Bullini, 1988; *Anisakis nascettii* Mattiucci, Paoletti & Webb, 2009; and *Anisakis berlandi* Mattiucci, Cipriani, Webb, Paoletti, Marcer, Bellisario, Gibson & Nascetti, 2014. All are parasites of different aquatic mammals (ziphiids, delphinids, sperm whales, or a wide array of delphinoid odontocetes and mysticetes) (Mattiucci & Nascetti, 2008; Mattiucci et al., 2014; Cabrera-Gil et al., 2018).

Previously, *Anisakis* had two subgenera, *Anisakis* and *Skrjabinisakis*, based on the shape and length of the ventriculus and male spicules, but few researchers used the subgenera (Takano & Sata, 2022). *Skrjabinisakis* was elevated to genus level by Safonova et al. (2021) based on the intraspecific genetic distances of ITS sequences; *Skrjabinisakis physeteris* (Baylis, 1923), *Skrjabinisakis brevispiculata* (Dollfus, 1966), *Skrjabinisakis paggiae (*Mattiucci, Nascetti, Dailey, Webb, Barros, Cianchi & Bullini, 2005) and *Skrjabinisakis schupakovi* (Mozgovoy, 1951) were included in this genus. Additionally, other studies using different genetic markers reaffirmed the validity of *Skrjabinisakis* (Takano & Sata, 2022; Bao et al., 2022; Chero et al., 2023).

In their study, Safonova et al. (2021) revalidated the generic status of *Peritrachelius*, relocating *A. typica* to the genus (*Peritrachelius typicu*). Takano & Sata (2022) and Chero et al. (2023), based on molecular data, did not assign *A*. *typica* to *Peritrachelius* since the species was aligned in *Anisakis s.s*., with similar phylogenetic relationships for *A*. *simplex s.s*. and *A*. *typica* observed by cox1 sequences. Mostafa et al. (2020) characterize *A*. *simplex s.s*., *A*. *pegreffii*, and *A*. *typica* as non-monophyletic groups; therefore, the use of isolated cox1 sequences may be inadequate for the reconstruction of phylogenetic relationships between *Anisakis* species.

The presence of parasites in fish is a challenge for Brazilian researchers, given the great territorial diversity, the number of fish species not yet catalogued, and the small number of taxonomists working in this group (Pavanelli et al., 2013). According to Shamsi (2021), the ability to identify parasite taxa down to species is especially important for resolving questions about biological diversity. However, training opportunities in parasite taxonomy are rare and increasingly decreasing.

Currently, checklists dealing with parasitic nematodes of fish in the Americas are scattered, geographically limited to a local scale, and/or mixed with other groups of metazoans (McDonald & Margolis, 1995; Garrido-Olvera et al., 2006; Luque et al., 2011, 2016; Arai & Smith, 2016; Santos et al., 2016; Lehun et al., 2020; Reis et al., 2021; Ramallo & Ailán-Choke, 2022; Pereira & González-Solís, 2022).

Epidemiological data on *Anisakis* spp. larvae provide elements for analysis and predictions of consumer exposure risk regarding the presence of these nematodes in commercial fish species, which represent a potential threat to the consumer (Cipriani et al., 2024). Furthermore, anisakiosis is a serious public health problem worldwide, as it is one of the most serious infections transmitted from fish to humans (Shamsi, 2021). Thus, this study aims to compile data on the morphology, morphometry, and parasite ecology of third-stage larvae (L3) of *Anisakis* recovered from fish in Brazil, in addition to gathering and analyzing retrospective data on the Brazilian ichthyofauna that hosts *Anisakis* sp. larvae.

## Material and Methods

This is a descriptive systematic review of scientific literature. We searched for relevant publications using internet search engines such as Medline, PubMed, Science Direct, Redalyc, Google, and Google Scholar and other databases (Web of Science, Biological Abstracts, Helminthological Abstracts, and Aquatic Science and Fisheries Abstracts). Additionally, we examined the reference lists of identified articles and also checked all available published lists of species (Vicente et al., 1985; Moravec, 1998; Vicente & Pinto, 1999; Eiras et al., 2010, 2016; Luque et al., 2011) to locate fish reported as infected by *Anisakis* spp. in Brazil. The following keywords were used during the electronic literature search: “fish nematodes”, “zoonosis”, “*Anisakis*”, “*Skrjabinisakis”*, ‘Anisakidae’, “human health” and “Brazil”. This retrospective study encompassed articles published in Brazil between 1983 and 2023. The collected data is presented in the results section.

The taxonomic status partially follows the systematic arrangement based on molecular data by Safonova et al. (2021). Host species are presented, followed by their predominant habitat (marine-MAR, freshwater-FW, or brackish water-BW) and site of infection; when possible, they are grouped. Localities are presented in alphabetical order of the Brazilian states and coast (AC-Acre, AM-Amazonas, BC-Brazilian coast, Bahia, CE-Ceará, MA-Maranhão, PA-Pará, PR-Paraná, RJ-Rio de Janeiro, RN-Rio Grande do Norte, and RS-Rio Grande do Sul) and records in chronological sequence.

Fish species names have been updated according to Froese & Pauly (2024). Nematode species names have been updated according to recent literature, but inclusion in parasite or host lists does not imply that the authors necessarily agree with their validity.

## Results

### Morphological data

Morphologically, we have different morphospecies assigned to third-stage larvae of *Anisakis* in Brazil, namely: *A*. *simplex*, *A*. *typica*, *A*. *pegreffii*, *Anisakis* sp., *Anisakis* sp. larva type I sensu Berland (1961), *Skrjabinisakis physeteris*, and *S*. *brevispiculata*. The morphological, morphometric, and prevalence characteristics of third-stage larvae (L3), in addition to the record of hosts occurring in Brazil, are presented below.

Family Anisakidae Raillet & Henry, 1912

Genus *Anisakis* Dujardin, 1945

**Figure 1:**
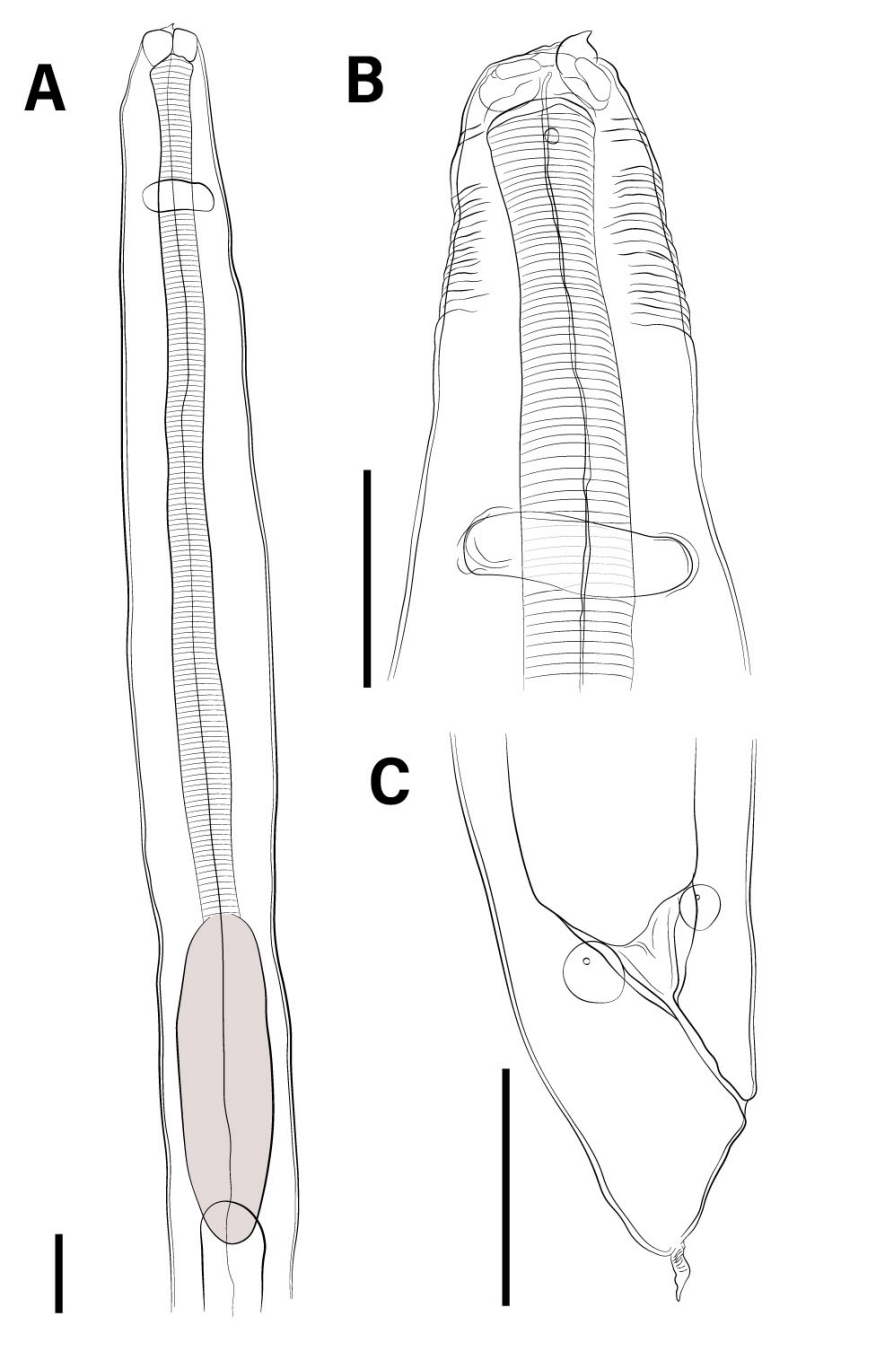
Drawings of L3 of *Anisakis* sp. parasite in *Propimelodus eigenmanni*, Brazilian fish: The scale bars in **A**, **B** and **C** = 100μm.

**Figure 2:**
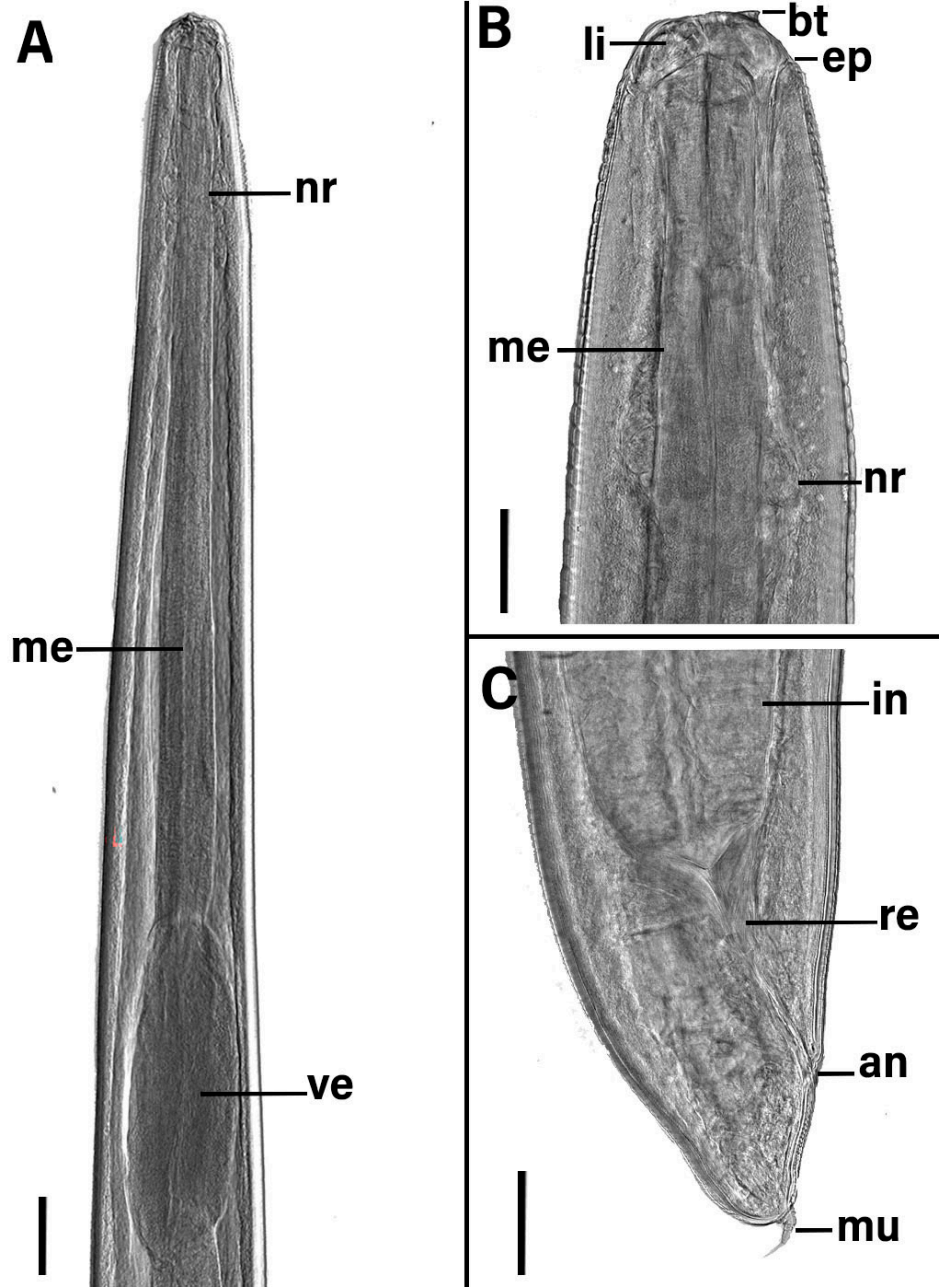
Photomicrographs of L3 *Anisakis* sp. parasite in *Propimelodus eigenmanni*, Brazilian fish: **(A)** Anterior end, showing nerve ring (nr), muscular esophagus (me) and ventriculus (ve); **(B)** Detail of anterior end, cuticle with delicate transversal striations, lips (li) larval tooth (lt), excretory pore (ep), muscular esophagus (me) and nerve ring (nr); **(C)** Posterior portion, showing end portion of the intestine (in), rectum (re) and anus (an), the tail with mucron (mu). The scale bars in A = 100μm, and B and C = 50μm.

**Figure 3:**
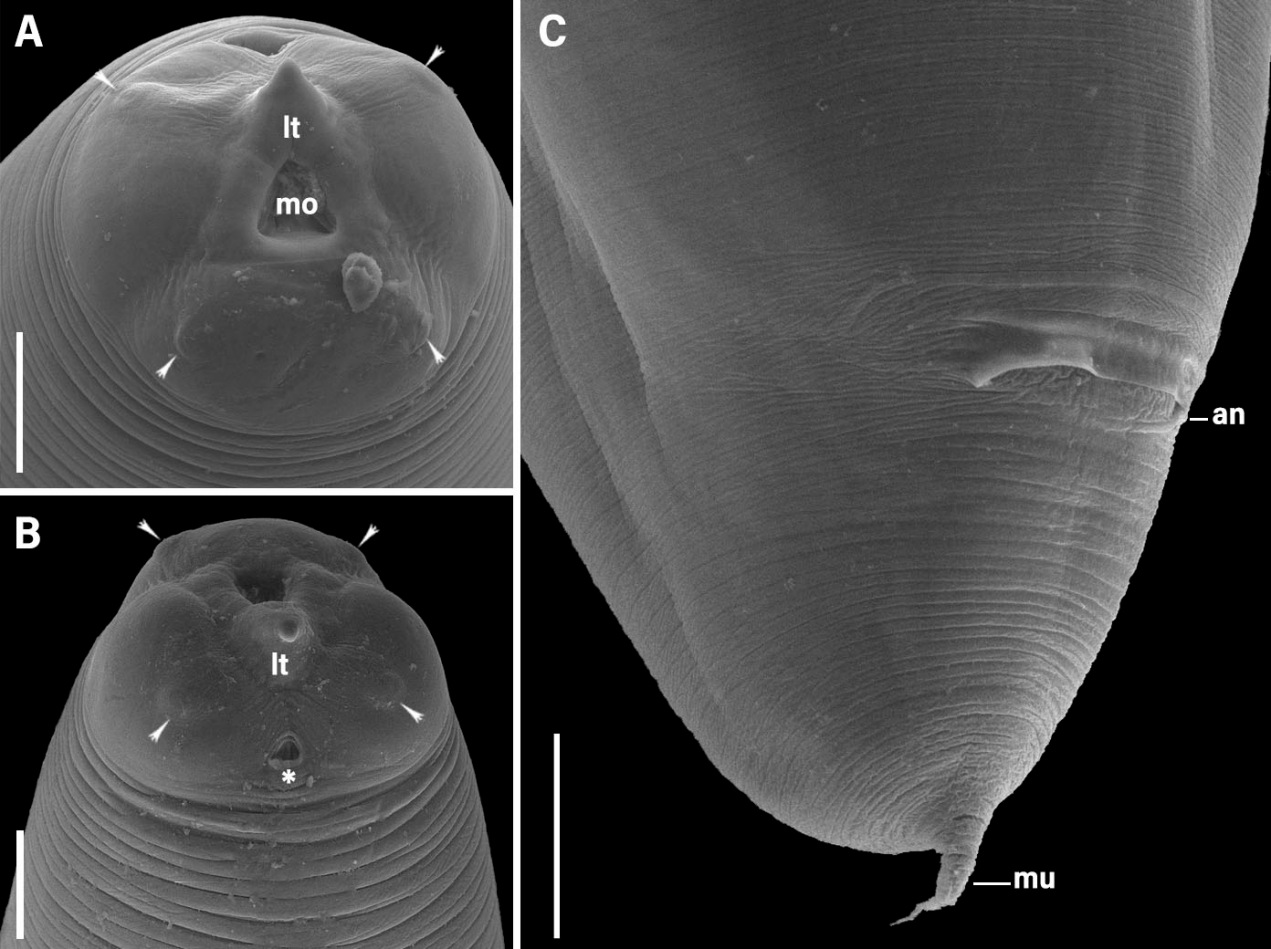
Scanning electron micrographs of L3 *Anisakis* sp. parasite in *Propimelodus eigenmanni*, Brazilian fish: **(A)** Cephalic region showing evidence cuticle with transversal striations, three lips, four papillae (arrowhead), mouth opening (mo), provided with larval tooth (lt) and excretory pore (ep); **(B)** Side view of cephalic region with papillae (arrowhead), larval tooth (lt) and excretory pore (*); **(C)** Posterior portion, anus (an), the tail with mucron (mu). The scale bars in A, B, C = 20μm.

*Anisakis* sp. (third stage larvae - L3) (Figures 1, 2 and 3)

Cuticle with fine transverse striations. Cephalic extremity with three poorly developed lips, one dorsal and two ventrolateral. Dorsal lip containing a pair of papillae and ventrolateral lips with one papilla and one amphid. Larval tooth below the oral opening, between the two ventrolateral lips. Excretory pore opening below the larval tooth. Deirids inconspicuous. Ventriculus longer than wide. Two spherical rectal glands. Conical tail and mucron present. The morphometric data of *Anisakis* larvae occurring in fish from Brazilian waters are presented in Table 1.

**Table 1:** Morphological and morphometric comparison of third-stage larvae of *Anisakis* collected in fish from Brazilian waters. (Measurements in millimeters; the parameter number of buds is given in amplitude).

**Character**		**Third stage larvae the *Anisakis* spp.**														
**Hosts larvae**		***Plagioscion squamosissimus***			***Priacanthus arenatus***			***Cichla monoculus***			***Plagiosciom squamosissimus***				***Lopholatilus villarii***	
**Length**		12.1−13.4			20.86−28.68			9.80−17.05			5.69−11.80				23.63	
**Width**		0.27−0.36			0.42−0.56			0.19−0.35			0.093−0.16				0.38	
**Nerve ring**		−			0.28−0.45			0.19−0.21			0.13−0.21				0.30	
**Esophagus ^L^**		0.98−1.40			1.42−2.20			0.81−1.18			0.69−1.05				1.75	
**Ventriculus ^L^**		0.36−0.68			0.30−1.00			0.28−0.47			0.18−0.41				0.70	
**Ventriculus ^W^**		0.12−0.20			0.20−0.30			0.09−0.17			0.052−0.13				0.20	
**Tail**		0.07−0.10			0.08−0.15			0.06−0.24			0.049−0.12				0.10	
**Mucron**		0.02−0.04			0.008−0.032			Present			Present				0.025	
**Number of specimens**		40			15			-			10				1	
**Reference**		Fontenelle et al. (2016)			Kuraiem et al. (2016)			Santana et al. (2017)			Gomes (2017)				Silva et al. (2017)	
**Character**		**Third stage larvae the *Anisakis* spp.**														
**Hosts larvae**		***Trichiurus lepturus***			***Prionotus punctatus***			***Ageneiosus ucayalensis***			***Lophius gastrophysus***			***Pygocentrus nattereri***		
**Length**		22.80−35.95			3.51−8.40			11.8−15.9			17.61−26.49			19−25		
**Width**		0.028−0.035			0.08−0.56			0.17−0.23			0.47−0.68			0.54−0.63		
**Nerve ring**		−			0.04−0.06			0.23−0.24			0.20−0.25			0.15−0.19		
**Esophagus ^L^**		1.37−1.76			0.34−0.74			−			1.06−2.01			1.90−2.60		
**Ventriculus ^L^**		0.065−0.088			0.12−0.37			0.36−0.46			0.57−0.75			0.50−0.60		
**Ventriculus ^W^**		0.007−0.016			0.03−0.11			0.11−0.15			0.15−0.23			0.40−0.50		
**Tail**		0.007−0.013			0.12−0.19			0.17			0.09−0.14			0.15−0.19		
**Mucron**		−			−			Present			Present			Present		
**Number of specimens**		−			8			−			9			10		
**Reference**		Barros & Amato (1993)			Bicudo et al. (2005a)			Giese (2010)			Vieira et al. (2012) a			Morais (2012), Morais et al. (2019)		
**Character**	**Third stage larvae the *Anisakis* spp.**					**Third stage larvae the *Anisakis simplex***										**Third stage larvae the *Anisakis typica***
**Hosts larvae**	***Lutjanus analis*** b		***Hypophthalmus marginatus***			***Paralichthys isosceles***			***Cynoscion guatucupa***			***Pseudopercis numida***				***Trichiurus lepturus***
**Length^a^**	20		9.32−13.87			15.3−16			22.25−23.50			−				15.34−22.43
**Width**	−		0.27−0.32			0.35−0.37			0.42−0.45			−				0.35−0.60
**Nerve ring**	−		0.10−0.25			0.26−0.28			0.30−0.35			−				−
**Esophagus ^L^**	1.5		0.74−1.10			1.53−1.62			1.80−1.90			1.52				1.10−1.81
**Ventriculus ^L^**	0.50		0.33−0.42			0.55−0.60			0.85−0.94			0.62				0.50−0.76
**Ventriculus ^W^**	−		0.13−0.16			0.22−0.25			0.30−0.34			−				−
**Tail**	−		0.10−0.75			0.07−0.08			0.10−0.15			0.09				0.08−0.20
**Mucron**	Present		Present			0.02−0.03			0.015−0.025			Present				Present
**Number of specimens**	56		3			−			−			1				12
**Reference**	Alves et al. (2020)		Cárdenas et al. (2021)			Felizardo et al. (2009)			Fontenelle (2013), Fontenelle et al. (2013)			Oliveira (2015)				Borges et al. (2012)
**Character**	**Third stage larvae the *Anisakis typica***												**Third stage larvae the *Skrjabinisakis brevispiculata***			
**Hosts larvae**	***Paralichthys patagonicus***			***Xystreurys rasile***			***Zenopsis conchifer***			***Micropogonias furnieri***			***Pinguipes brasilianus***			
**Length**	22.40−24.95			21.05−24.97			−			16			−			
**Width**	0.40–0.42			0.40–0.47			0.22−0.25			0.50			0.35			
**Nerve ring**	0.20–0.25			0.28–0.32			0.13−0.14			0.43			0.16			
**Esophagus** **L**	1.75–1.85			1.50–1.70			1.12−1.68			1.6			1.45			
**Ventriculus ^L^**	0.67−0.82			0.89–0.94			0.44−0.49			0.6			0.41			
**Ventriculus** **W**	0.15−0.27			0.25−0.30			−			−			−			
**Tail**	0.80−0.12			0.09–0.13			0.18−0.19			0.5			0.10			
**Mucron**	0.005−0.020			0.005−0.010			Present			Present			Absent			
**Number of specimens**	5			7			3^c^			1			1			
**Reference**	Fonseca et al. (2016)			Fonseca et al. (2016)			Sardella & Luque (2016), Sardella (2017)			Di Azevedo & Iñiguez (2018)			Sardella & Luque (2016), Sardella (2017)			

L length;

W width;

a Identified as *Anisakis* sp. larva type I sensu Berland (1961);

b Other hosts cited by Alves et al. (2020): *Lutjanus jocu, Lutjanus synagris, Lutjanus vivanus, Ocyurus chrysurus*;

c Based on 3 specimens 1 collected from *Zenopsis conchifer* and 2 collected from *Auxis thazard*.

### Biodiversity data on fish that harbor third-stage *Anisakis* larvae

**Figure 4:**
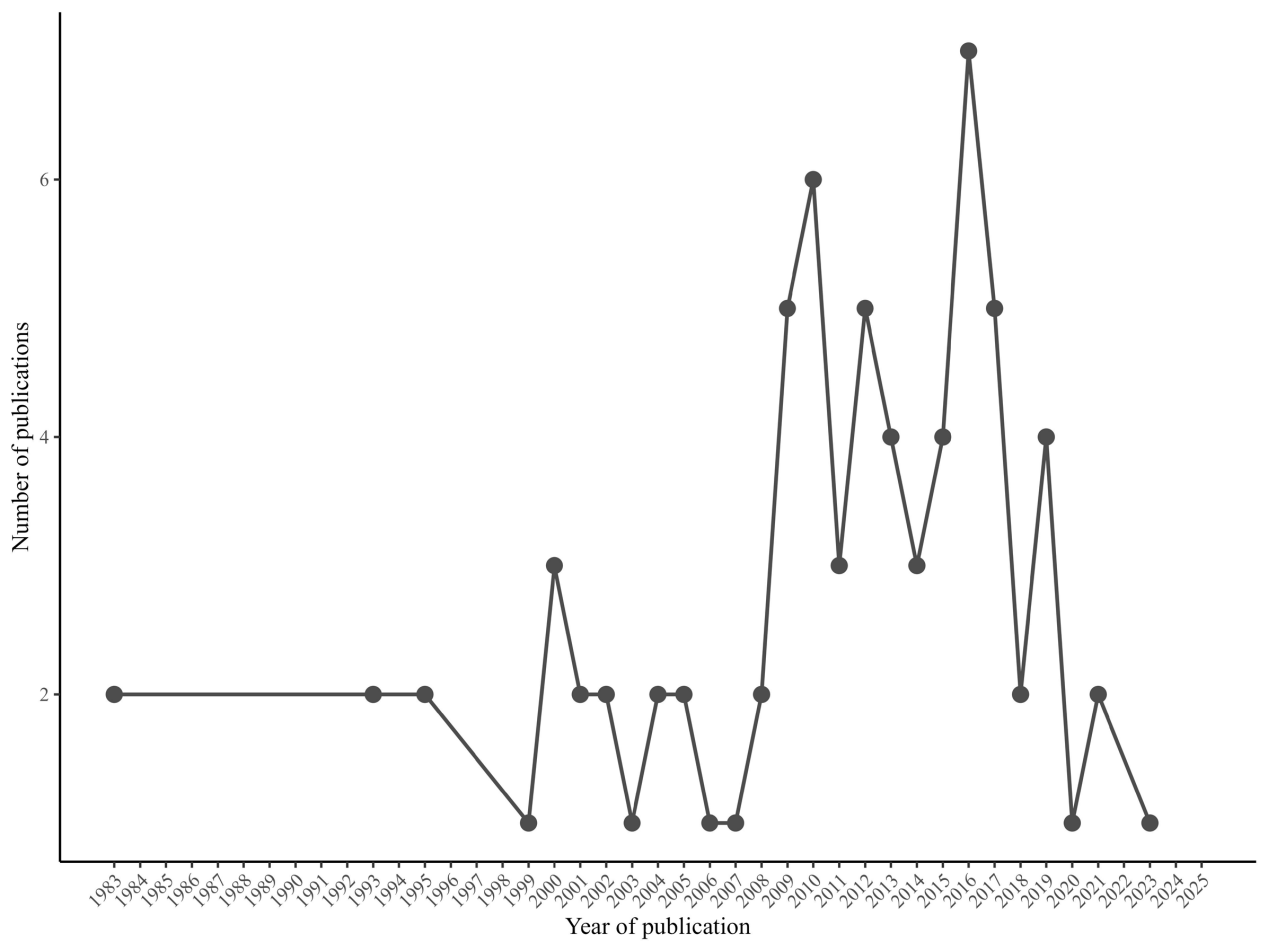
Number of papers published per year on third-stage larvae of *Anisakis* spp. parasitic on fish from Brazilian waters.

**Figure 5:**
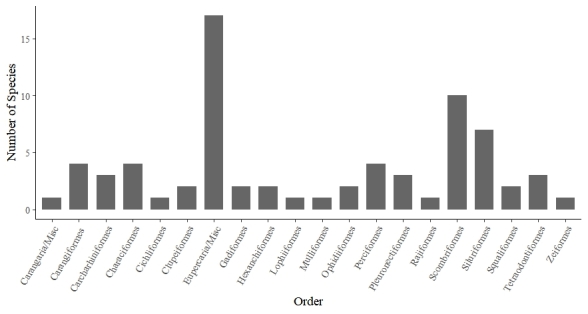
Diversity of Brazilian fishes species parasitized by stage larvae of *Anisakis* spp. distributed by order.

**Figure 6:**
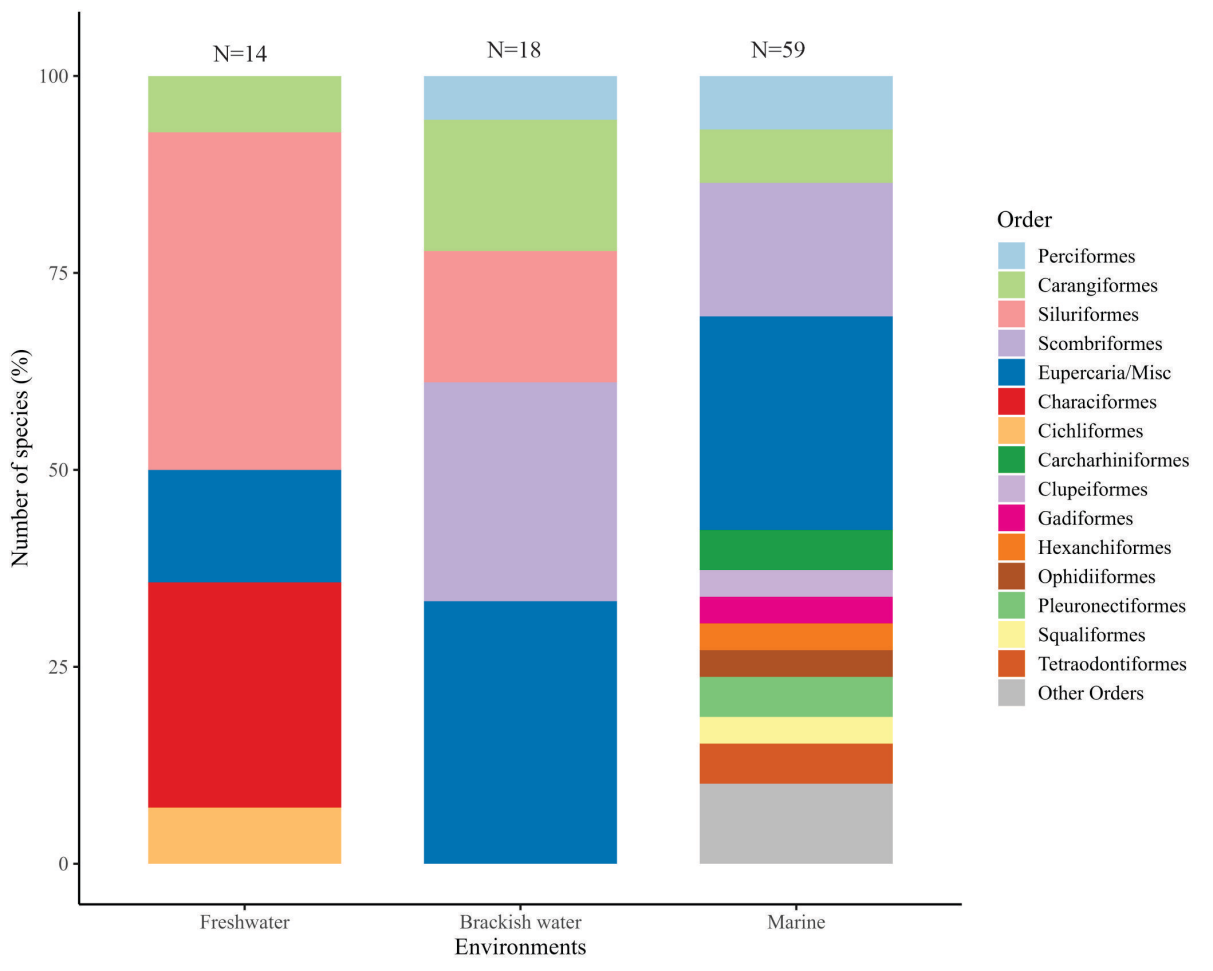
Distribution of Brazilian fishes species parasitized by stage larvae of *Anisakis* spp. in different habitats (freshwater, brackish and marine), grouped by order.

The biogeographic study of fish species presents in the Brazilian ichthyofauna that are reported as intermediate hosts of *Anisakis* L3 is presented in a retrospective study obtained from 75 works published between 1983 and 2023, including articles, dissertations, and theses (Figure 4). This study resulted in 18 orders and two groups/misc (14 orders of Osteichthyes and four of Chondrichthyes; groups Eupercaria/misc and Carangaria/misc) (Figure 5); with 40 families (35 of Osteichthyes and 5 of Chondrichthyes), 60 genera (53 of Osteichthyes and 7 of Chondrichthyes), and 69 species (62 of Osteichthyes and 7 of Chondrichthyes) (Table 2) distributed in three aquatic habitats. Predominantly marine fish were the most prevalent, with 62% of the fish in this environment parasitized by *Anisakis* larvae (Figure 6).

**Table 2:** Check list of records of third-stage larvae of *Anisakis* spp. in fishes from Brazilian waters.

**Order/ Family/ Host** a	**Site of infection** b	**Locality** c	**Reference** d
**Carangaria/misc**			
**Family Sphyraenidae**			
*Sphyraena guachancho* Cuvier, 1829^MAR^	ME	RJ	Luque & Poulin (2004), Luque et al. (2011), Eiras et al. (2016)

**Order Carangiformes**			
**Family Carangidae**			
*Caranx latus* Agassiz, 1831^MAR, FW, BW^	ME	RJ	Luque et al. (2000, 2011), Luque & Poulin (2004), Eiras et al. (2016)
*Selene setapinnis* (Mitchill, 1815)^MAR, BW^	ME	RJ	Cordeiro & Luque (2004), Luque & Poulin (2004), Luque et al. (2011), Eiras et al. (2016)
*Trachurus lathami* Nichols, 1920^MAR^	ME	RJ	Alves & Gonçalves (2012), Eiras et al. (2016)^e^
**Family Coryphaenidae**			
*Coryphaena hippurus* Linnaeus, 1758^MAR, BW^	NS	BC	Barros & Cavalcanti (1998), Eiras et al. (2016)

**Order Characiformes**			
**Family Acestrorhynchidae**			
*Acestrorhynchus falcatus* (Bloch, 1794)^FW^	GT	AM	Murrieta-Morey & Oliveira Malta (2018), Reis et al. (2021)
**Family Serrasalmidae**			
*Pygocentrus nattereri* Kner, 1858^FW^	IN	AM	Morais (2012), Morais et al. (2019), Reis et al. (2021)
*Serrasalmus altispinis* Merckx, Jégu & Santos, 2000^FW^	IN	AM	Murrieta-Morey & Oliveira-Malta (2016), Reis et al. (2021)
**Family Triportheidae**			
*Triportheus angulatus* (Spix & Agassiz, 1829)^FW^	NS	AM	Moreira et al. (2017), Reis et al. (2021)

**Order Cichliformes**			
**Family Cichlidae**			
*Cichla monoculus* Agassiz, 1831^FW^	IN, LI	AM	Santana et al. (2017)

**Order Clupeiformes**			
**Family Alosidae**			
*Brevoortia aurea* (Spix & Agassiz, 1829)^MAR^	ME	RJ	Tavares et al. (2004), Luque & Poulin (2004), Luque et al. (2011), Eiras et al. (2016)
**Family Engraulidae**			
*Engraulis anchoita* Hubbs & Marini, 1935^MAR^	NS	UL	Eiras et al. (2016) **^e^**

**Eupercaria/misc**			
**Family Latilidae**			
*Lopholatilus villarii* Miranda Ribeiro, 1915^MAR^	INS	RJ	Silva et al. (2017)
**Family Lutjanidae**			
*Lutjanus analis* (Cuvier, 1828)^MAR, BW^	ME	BC	Alves et al. (2020)
*Lutjanus campechanus* (Poey, 1860)^MAR^	NS	UL	Eiras et al. (2016)
*Lutjanus jocu* (Bloch & Schneider, 1801)^MAR, BW, FW^	TI	BC	Alves et al. (2020)
*Lutjanus purpureus* (Poey, 1866)^MAR^	ME, STS, ST, INS, IN, GO	RN	Barros & Cavalcanti (1998), Cavalcanti (2010)**^e^**
*Lutjanus synagris* (Linnaeus, 1758)^MAR^	TI	BC	Alves et al. (2020)
*Lutjanus vivanus* (Cuvier, 1828)^MAR^	TI	BC	Alves et al. (2020)
*Ocyurus chrysurus* (Bloch, 1791)^MAR^	TI	BC	Alves et al. (2020)
**Family Priacanthidae**			
*Priacanthus arenatus* Cuvier, 1829^MAR^	CA, ST, LI, AM	RJ	Ferreira (2008), Kuraiem (2015), Kuraiem et al. (2016), Eiras et al. (2016)
**Family Sciaenidae**			
*Cynoscion guatucupa* (Cuvier, 1830)^MAR^	ME	RJ	Fontenelle (2013)**^e^**, Fontenelle et al. (2013), Eiras et al. (2016)**^e,^**^f^
*Cynoscion* sp. ^MAR^	NS	SP	Vaz (2010), Eiras et al. (2016)
*Macrodon ancylodon* (Bloch & Schneider, 1801)^MAR, BW^	NS	UL	Luque et al. (2011), Eiras et al. (2016)
*Micropogonias furnieri* (Desmarest, 1823)^MAR, BW^	IN, ME	RJ, CE	Luque et al. (2010, 2011), Eiras et al. (2016), Di Azevedo & Iñiguez (2018)^g^,
*Nebris microps* Cuvier, 1830^MAR, BW^	ME	UL	Luque et al. (2011), Eiras et al. (2016)
*Plagioscion squamosissimus* (Heckel, 1840)^FW^	ME, IN, AC	PA	Rodrigues et al. (2015), Fontenelle et al. (2016), Gomes (2017), Reis et al. (2021)
*Umbrina canosai* Berg, 1895 ^MAR, BW^	ME	RJ	Canel et al. (2019) **^e^**
**Family Sparidae**			
*Pagrus pagrus* (Linnaeus, 1758)^MAR^	ME, LI, HC, MU	RJ	Paraguassú et al. (2002); Luque & Poulin (2004), Ferreira (2008), Muniz-Pereira et al. (2009), Saad & Luque (2009), Luque et al. (2011), Mattos (2012), Figueiredo Jr et al. (2013b**^e^**, 2016), Mattos et al. (2014), Soares (2014), Soares et al. (2014), Eiras et al. (2016)

**Order Gadiformes**			
**Family Merlucciidae**			
*Merluccius hubbsi* Marini, 1933^MAR^	ME	UL	Luque et al. (2011), Eiras et al. (2016)
**Family Phycidae**			
*Urophycis mystacea* Miranda Ribeiro, 1903^MAR^	ME	RJ	Luque & Poulin (2004), Luque et al. (2011), Eiras et al. (2016)

**Order Lophiiformes**			
**Family Lophiidae**			
*Lophius gastrophysus* Miranda Ribeiro, 1915^MAR^	ME, AC	RJ	Vieira et al. (2012)^h^, Knoff et al. (2013)**^e^**, Eiras et al. (2016)**^e,f^**

**Order Mulliformes**			
**Family Mullidae**			
*Mullus argentinae* Hubbs & Marini, 1933^MAR^	ME	RJ	Luque et al. (2002, 2011), Luque & Poulin (2004), Eiras et al. (2016)

**Order Ophidiiformes**			
**Family Ophidiidae**			
*Genypterus blacodes* (Forster, 1801)^MAR^	NS	UL	Eiras et al. (2016) **^e,f,^** i
*Genypterus brasiliensis* Regan, 1903^MAR^	MU, ME, STS, INS, OV, ST, IN	RJ	Knoff et al. (2003, 2004, 2007)**^e,f,i^**, Padovani et al. (2005), Luque et al. (2011)**^e,i^**, Mattos (2012), Figueiredo et al. (2013b**^e^**, 2016), Mattos et al. (2014)

**Order Perciformes**			
**Family Percophidae**			
*Percophis brasiliensis* Quoy & Gaimard, 1825^MAR^	ME	RJ	Luque & Poulin (2004), Luque et al. (2011), Eiras et al. (2016)
**Family Pinguipedidae**			
*Pinguipes brasilianus* Cuvier, 1829^MAR^	ST	RJ	Sardella & Luque (2016)^j^, Sardella (2017)**^j^**
*Pseudopercis numida* Miranda Ribeiro, 1903^MAR^	ME	RJ	Luque et al. (2008, 2011), Oliveira (2015)**^g^**, Eiras et al. (2016)
**Family Triglidae**			
*Prionotus punctatus* (Bloch, 1793)^MAR, BW^	ME, LI	RJ	Luque & Poulin (2004), Bicudo et al. (2005a,b), Luque et al. (2011), Eiras et al. (2016)

**Order Pleuronectiformes**			
**Family Paralichthyidae**			
*Paralichthys isosceles* Jordan, 1891^MAR^	AC, IN, STS, ME	RJ	Luque & Poulin (2004), Felizardo et al. (2009)**^e,f^**, Luque et al. (2011)**^e^**, Alarcos et al. (2016), Eiras et al. (2016)**^e,f^**
*Paralichthys patagonicus* Jordan, 1889^MAR^	ST, IN, LI, AC,	RJ	Fonseca et al. (2016) **^g^**
*Xystreurys rasilis* (Jordan, 1891)^MAR^	ST, IN, LI, AC,	RJ	Fonseca et al. (2016) **^g^**

**Order Scombriformes**			
**Family Gempylidae**			
*Thyrsitops lepidopoides* (Cuvier, 1832)^MAR^	NS	RJ	Alves & Domingues (2015)
**Family Pomatomidae**			
*Pomatomus saltatrix* (Linnaeus, 1766) ^MAR, BW^	AC, ST, IN	RJ	Rego et al. (1983)**^e^**, Vicente et al. (1985)**^e^**, Luque & Chaves (1999), Luque & Poulin (2004), Luque et al. (2011), Eiras et al. (2016)
**Family Trichiuridae**			
*Trichiurus lepturus* Linnaeus, 1758^MAR, BW^	ME, AC, COS, MU	RJ	Barros & Amato (1993), Marques et al. (1995), São Clemente et al. (1996), Silva et al. (2000a,b), Luque & Poulin (2004), Luque et al. (2011), Borges et al. (2012)**^g^**, Mattos (2012), Figueiredo et al. (2013b**^e^**, 2016), Mattos et al. (2014), Eiras et al. (2016)**^f,g^**
**Family Scombridae**			
*Auxis thazard* (Lacepède, 1800)^MAR^	ME, IN	BC, RJ	Iñiguez et al. (2009)**^g,i^**, Luque et al. (2011)**^g,i^**, Eiras et al. (2016)**^g,i^**, Sardella & Luque (2016)**^g^**, Sardella (2017)**^g^**
*Euthynnus alletteratus* (Rafinesque, 1810)^MAR, BW^	ME	RJ	Luque & Poulin (2004), Alves & Luque (2006), Luque et al. (2011), Eiras et al. (2016)
*Katsuwonus pelamis* (Linnaeus, 1758)^MAR^	ME	RJ	Alves & Luque (2006), Luque et al. (2011), Eiras et al. (2016)
*Scomberomorus cavalla* (Cuvier, 1829)^MAR^	STS	RJ	Dias et al. (2011), Luque et al. (2011), Eiras et al. (2016)
*Scomber colias* Gmelin, 1789^MAR^	ME, ST, PC, IN, LI, HE, SB	RJ	Rego & Santos (1983), Vicente et al (1985)**^e^**, Abdallah et al. (2002), Alves et al. (2003), Luque & Poulin (2004), Luque et al. (2011), Eiras et al. (2016)
*Scomber scombrus* Linnaeus, 1758^MAR, BW^	ME	RJ	Luque & Poulin (2004), Alves & Luque (2006), Luque et al. (2011), Eiras et al. (2016)
*Thunnus thynnus* (Linnaeus, 1758) ^MAR, BW^	NS	BC	Luque et al. (2011)**^g,^**^k^, Eiras et al. (2016)**^g,k^**

**Order Siluriformes**			
**Family Ariidae**			
*Bagre bagre* (Linnaeus, 1766)^MAR, BW^	ME	UL	Luque et al. (2011),
**Family Auchenipteridae**			
*Ageneiosus ucayalensis* Castelnau, 1855^FW^	STS, IN, LI	PA	Giese (2010)
**Family Doradidae**			
*Oxydoras niger* (Valenciennes, 1821)^FW^	NS	PA	Rodrigues et al. (2015)
**Family Pimelodidae**			
*Brachyplatystoma filamentosum* (Lichtenstein, 1819)^FW, BW^	NS	PA	Rodrigues et al. (2015)
*Brachyplatystoma rousseauxii* (Castelnau, 1855)^FW^	ME	PA	Salgado (2011)
*Hypophthalmus marginatus* Valenciennes, 1840^FW^	ME, LI	MA	Cárdenas et al. (2021)
*Pimelodus blochii* Valenciennes, 1840^FW, BW^	NS	AC	Cavalcante et al. (2020), Reis et al. (2021)

**Order Tetraodontiformes**			
**Family Balistidae**			
*Balistes vetula* Linnaeus, 1758^MAR^	ME	RJ	Alves et al. (2005), Muniz-Pereira et al. (2009), Luque et al. (2011), Eiras et al. (2016),
**Family Monacanthidae**			
*Aluterus monoceros* (Linnaeus, 1758)^MAR^	ME	RJ	Dias et al. (2010), Luque et al. (2011), Eiras et al. (2016)
**Family Tetraodontidae**			
*Colomesus psittacus* (Bloch & Schneider, 1801)^MAR^	ME, CC	PA	Giese et al. (2023)

**Order Zeiformes**			
**Family Zeidae**			
*Zenopsis conchifer* (Lowe, 1852)^MAR^	ST	RJ	Sardella & Luque (2016)**^g^**, Sardella (2017)**^g^**

**Subclass Elasmobranchii**			
**Order Carcharhiniformes**			
**Family Carcharhinidae**			
*Carcharhinus signatus* (Poey, 1868)^MAR^	ST, SV	PR	Knoff et al. (2001), Muniz-Pereira et al. (2009), Luque et al. (2011), Eiras et al. (2016)
**Family Triakidae**			
*Galeorhinus galeus* (Linnaeus, 1758)^MAR^	SV	RS	Knoff et al. (2001), Luque et al. (2011), Eiras et al. (2016)
*Mustelus canis* (Mitchill, 1815)^MAR^	SV	RS	Knoff et al. (2001), Luque et al. (2011), Eiras et al. (2016)

**Order Hexanchiformes**			
**Family Hexanchidae**			
*Heptranchias perlo* (Bonnaterre, 1788)^MAR^	SV	PR	Knoff et al. (2001), Luque et al. (2011), Eiras et al. (2016)
*Hexanchus griseus* (Bonnaterre, 1788)^MAR^	SV	PR	Knoff et al. (2001), Luque et al. (2011), Eiras et al. (2016)

**Order Rajiformes**			
**Family Rajidae**			
*Dipturus trachyderma* (Krefft & Stehmann, 1975)^MAR^	SV	PR	Knoff et al. (2001), Muniz-Pereira et al. (2009), Luque et al. (2011), Eiras et al. (2016)

**Order Squaliformes**			
**Family Squalidae**			
*Squalus megalops* (Macleay, 1881)^MAR^	SV	RS	Knoff et al. (2001), Luque et al. (2011), Eiras et al. (2016)
*Squatina* sp.^MAR^	SV	RS	Knoff et al. (2001), Luque et al. (2011)

a abbreviations: Host species are given followed by their predominant habitat (marine = MAR, freshwater = FW or brackish water = BW);

b abbreviations: Site of the infection; when possible these are grouped (AC = abdominal cavity; AM = abdominal muscles; CA = caecum; CC = coelomic cavity, COS = coelomic serosa; GT = gastrointestinal tract; HE = heart; HC = hepatic capsule; IN = intestine; INS = intestinal serosa; LI = liver; ME = mesenteries; Mu = muscle; OV = ovary; STS = stomach serosa; PC = pyloric caeca; SB = swim bladder; ST = stomach; SV = spiral valve; TI = Tissues and NS = not specified);

c localities of occurrence of third-stage larvae *Anisakis*, are presented in alphabetical order of Brazilian states (AC = Acre; AM = Amazonas; BC = Brazilian coast; CE = Ceará; MA = Maranhão; PA = Pará; PR = Paraná; RJ = Rio de Janeiro; RN = Rio Grande do Norte; RS = Rio Grande do Sul; SP= São Paulo and UL= Unspecified locations);

d records bibliographical by host in chronological sequence;

e Morphospecies identified as *Anisakis simplex*;

f Morphospecies identified as *Anisakis* sp.;

g Morphospecies identified as *Anisakis typica*;

h Morphospecies identified as *Anisakis* sp. larva type I sensu Berland (1961);

i Morphospecies identified as *Skrjabinisakis physeteris*;

j Morphospecies identified as *Skrjabinisakis brevispiculata*;

k Morphospecies identified as *Anisakis pegreffii*.

**Figure 7:**
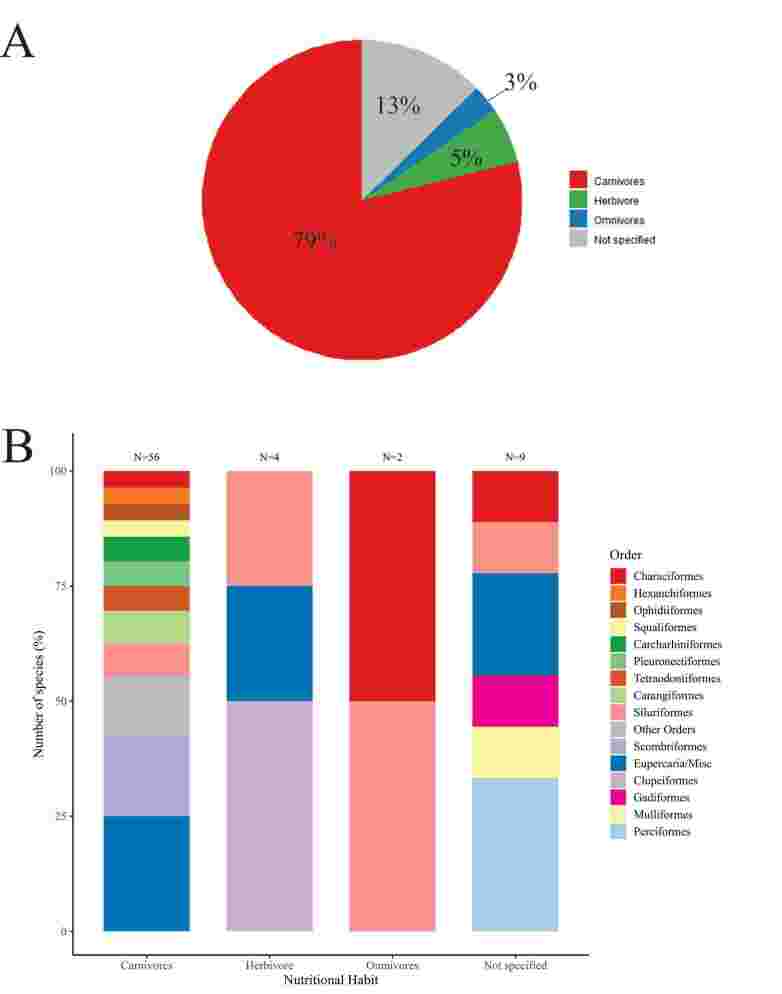
Distribution of Brazilian fishes species parasitized by third-stage larvae of *Anisakis* spp. according to feeding habits: (A) percentage of different feeding habits (carnivore, omnivore and herbivore); (B) number of species with different feeding habits grouped by order.

The fishes of the Eupercaria/Misc group were the most representative in diversity, with 5 families and 17 species hosting *Anisakis* larvae. Although they present low species diversity, the orders Cichliformes, Lophiiformes, Mulliformes, and Zeiformes demonstrated their importance as small-scale and industrial fishing resources. Of the 69 fish species analyzed, *Scomber colias* Gmelin, 1789 (*Scomber japonicus* Houttuyn, 1782 has had its distribution updated to the Indian Ocean and *Scomber colias* is present in the Atlantic Ocean) presented the highest number of infection sites for *Anisakis* larvae, with 55% of the total records in the literature citing the mesentery as the main site of larval occurrence; for Chondrichthyes fish, 100% presented parasitized spiral valves. In terms of diet, 79% of the species cited are carnivorous (Figure 7).

**Figure 8:**
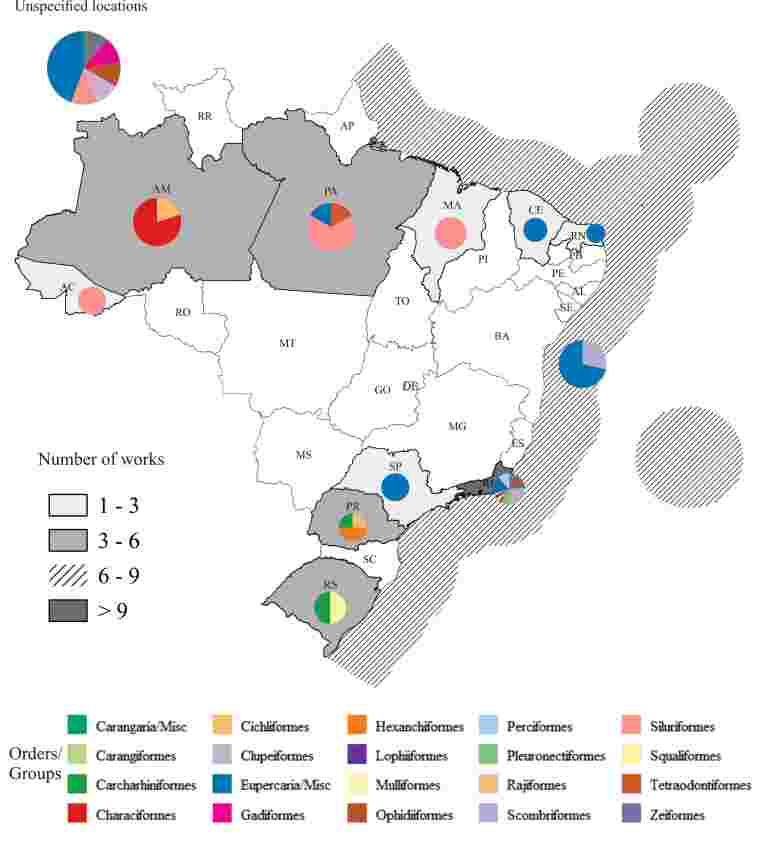
Distribution by state and Brazilian coast of third-stage larvae of *Anisakis* spp. parasitic on Brazilian fishes. The gray gradient indicates the number of papers per state, while pie charts show the diversity of parasitized fish orders. The exclusive economic zone is used to represent the “Brazilian coast”.

Regarding the distribution of hosts and information on available locations, the state of Rio de Janeiro ranks first in the number of records of fish with *Anisakis* larvae, followed by the states of Pará and Amazonas, respectively. *Anisakis* larvae infecting fish have also been recorded and are distributed in six other states, in addition to the records for fish caught on the Brazilian coast (Figure 8). Among the species with the highest number of scientific records for parasitism by L3 of *Anisakis*, there are two marine species, *Trichiurus lepturus* and *Pagrus pagrus* (Rego et al., 1983; Barros & Amato, 1993; Marques et al., 1995; São Clemente et al., 1996; Silva et al., 2000a, b; Paraguassú et al., 2002; Luque & Poulin, 2004; Ferreira, 2008; Muniz-Pereira et al., 2009; Saad & Luque, 2009; Luque et al., 2011; Mattos, 2012; Borges et al., 2012; Figueiredo et al., 2013b, 2016; Mattos et al., 2014; Soares, 2014; Soares et al., 2014; Eiras et al., 2016).

Among all the studies analyzed in this research, few species presented a prevalence of *Anisakis* above 50%; among these are the species *Lutjanus jocu* (71.42%), *Lutjanus purpureus* (75.51%), *Lutjanus vivanus* (86.27%), *Mullus argentinae* (66%), *Carcharhinus signatus* (60%), and *Hypophthalmus marginatus* (85.71%). *Plagioscion squamosissimus*, *Pimelodus blochii*, and *Hexanchus griseus* presented 100% prevalence of infection by *Anisakis* larvae. *Plagioscion squamosissimus* also presented the greatest range of infections, from 3 to 472 larvae per fish. Additional data on parasite prevalence for *Anisakis* larvae in Brazilian fish are presented in Table 3.

**Table 3:** Parasitological indices of infection of third-stage larvae of *Anisakis* spp. in fish from Brazilian waters.

**Order/ Family/ Host**	***n***	**P(%)**	**I^a^/ MI±SD**	**MA±SD**	**RI**	**Reference**
**Order Carangiformes**						
**Family Carangidae**						
*Caranx latus*	55	1.8	1	0.1	0.1	Luque et al. (2000)
*Selene setapinnis*	89	9.0	2.5±1.5	0.1±0.7	1.0-5.0	Cordeiro & Luque (2004)
*Trachurus lathami*	64	9.37	3.0±2.89	0.28±1.20	1.0-8.0	Alves & Gonçalves (2012)
**Family Corphaenidae**						
*Coryphaena hippurus*	18	11.1	41^a^	−	−	Barros & Cavalcanti (1998)

**Order Characiform**						
**Family Acestrorhynchidae**						
*Acestrorhynchus falcatus*	263	5.36	10±5.62	0.53±2.37	1.0-3.0	Murrieta-Morey & Oliveira Malta (2018)

**Family Serrasalmidae**						
*Pygocentrus nattereri*	355	8.73	159**^a^**/ 5.13±4.28	0.45	1-40	Morais (2012), Morais et al. (2019)
*Serrasalmus altispinis*	60	11.7	1.86±1.9	0.11	1.0-6.0	Murrieta-Morey & Oliveira-Malta (2016)
**Family Triportheidae**						
*Triportheus angulatus*	86	3.49	1.0	0.03±0.18	−	Moreira et al. (2017)

**Order Cichliformes**						
**Family Cichlidae**						
*Cichla monoculus*	38	13.15	2.6	0.34	−	Santana et al. (2017)

**Order Clupeiformes**						
**Family Alosidae**						
*Brevoortia aurea*	42	9.5	1.8±1.0	0.2±0.6	**−**	Tavares et al. (2004)

**Eupercaria/misc**						
**Family Latilidae**						
*Lopholatilus villarii*	31	3.2	2.0**^a^**	0.06	−	Silva et al. (2017)
**Family Lutjanidae**						
*Lutjanus analis*	69	20.28	15.28 (±36.27)	3.10 (±17.15)	−	Alves et al. (2020)
*Lutjanus jocu*	20	71.42	31.10 (±29.50)	22.21 (±28.55)	−	Alves et al. (2020)
*Lutjanus purpureus*	82	2.4	12^a^	−	−	Barros & Cavalcanti (1998)
	98	75.51	13.74	10.37	−	Cavalcanti (2010) b
*Lutjanus synagris*	27	17.39	3.25 (±2.87)	0.56 (±1.64)	−	Alves et al. (2020)
*Lutjanus vivanus*	51	86.27	27.88 (±25.01)	24.05 (±25.14)	−	Alves et al. (2020)
*Ocyurus chrysurus*	29	6.80	7±2.82	0.48±1.88	−	Alves et al. (2020)
**Family Priacanthidae**						
*Priacanthus arenatus*	30	20.0	5.0	1.0	−	Kuraiem (2015), Kuraiem et al. (2016)
**Family Sciaenidae**						
*Cynoscion guatucupa*	30	10.0	1.0	0.1	−	Fontenelle (2013)^b^, Fontenelle et al. (2013)
*Cynoscion* sp.	92	3.84	−	−	−	Vaz (2010)
*Micropogonias furnieri*	248	2.5	−	0.2±1.0	−	Luque et al. (2010)
	30	1.7	1.0	0.017	−	Di Azevedo & Iñiguez (2018) c
*Plagioscion squamosissimus*	10	10.0	−	−	−	Rodrigues et al. (2015),
	30	23.33	2.29±1.03	0.53±1.09	1-4	Fontenelle et al. (2016) d
	14	28.57	13.25±7.76	3.79±7.28	1-22	
	30	100	768**^a^**	−	3.0-472	Gomes (2017)
*Umbrina canosai*	36	11.11	−	0.14	0-2.0	Canel et al. (2019) **^b,^** e
	51	4.0	−	0.08	0-2.0	
**Family Sparidae**						
*Pagrus pagrus*	90	7.7	2.9±1.9	0.2±0.9	1.0-7.0	Paraguassú et al. (2002)
	36	5.56	1.0	0.06±0.24	−	Saad & Luque (2009)
	213	22.22	8.25	1.83	2.0-13.0	Mattos (2012), Mattos et al. (2014)
	100	40.0	12.0±8	5.0±10.0 2.38±7.37	1.0-65.0	Soares (2014), Soares et al. (2014)

**Order Lophiiformes**						
**Family Lophiidae**						
*Lophius gastrophysus*	36	22.2	−	0.53	−	Vieira et al. (2012) f
	87	1.14	1.0^a^	0.01	−	Knoff et al. (2013) **^b^**

**Order Mulliformes**						
**Family Mullidae**						
*Mullus argentinae*	100	66.0	5.7±7.4	3.8±6.6	1.0-378	Luque et al. (2002)

**Order Ophidiiformes**						
**Family Ophidiidae**						
*Genypterus brasiliensis*	55	21.8	11.7	−	−	Knoff et al. (2003)
	38	2.6	−	−	−	Knoff et al. (2004)
	74	1.35	4.0	0.05	1.0-4.0	Knoff et al. (2007) g
	74	1.35	8.4	1.13	1.0-15.0	Knoff et al. (2007) **^b^**
	74	1.35	5.9	0.81	1.0-23.0	Knoff et al. (2007)
	18	38.88	9.85	3.83	1.0-22.0	Mattos (2012), Mattos et al. (2014)

**Order Perciformes**						
**Family Pinguipedidae**						
*Pinguipes brasilianus*	30	3.3	1.0**^a^**	−	−	Sardella & Luque (2016)**^f^**, Sardella (2017)**^f^**
*Pseudopercis numida*	62	4.8	1.7±0.6	0.1±0.4	−	Luque et al. (2008)
	25	4.0	1.0**^a^**	1.0	−	Oliveira (2015) **^c^**
**Family Triglidae**						
*Prionotus punctatus*	80	17.5	1.6±1.45	0.29±0.86	1.0-6.0	Bicudo et al. (2005a,b)

**Order Pleuronectiformes**						
**Family Paralichthyidae**						
*Paralichthys isosceles*	60	5.0	1.0**^a^**	0.05	−	Felizardo et al. (2009) **^e^**
	38	2.6	−	0.03±0.2	−	Alarcos et al. (2016) h
	40	11.5	−	0.2±0.6	−	
*Paralichthys patagonicus*	36	11.1	1.25±0.5	0.13±2.82	1.0-2.0	Fonseca et al. (2016) i
*Xystreurys rasilis*	30	16.6	1.8±1.09	0.3±1.41	1.0-3.0	Fonseca et al. (2016) ^i^

**Order Scombriformes**						
**Family Gempylidae**						
*Thyrsitops lepidopoides*	55	7.3	1.75±1.5	0.13±0.6	1.0-4.0	Alves & Domingues (2015)
**Family Pomatomidae**						
*Pomatomus saltatrix*	60	3.3	−	−	−	Rego et al. (1983) **^e^**
	55	14.5	1.9	0.30	1.0−4.0	Luque & Chaves (1999)
**Family Trichiuridae**						
*Trichiurus lepturus*	217	2.77	402	−	−	Barros & Amato (1993)
	70	0.3	**−**	−	−	Marques et al. (1995)
	40	0.7	−	−	−	São Clemente et al. (1996)
	55	12.7	1.1±0.3	0.1±0.4	1.0-2.0	Silva et al. (2000b)
	64	20.31	−	−	1.0-10.0	Borges et al. (2012) **^g^**
	35	28.57	8.60	2.45	1.0-54.0	Mattos (2012), Mattos et al. (2014)
**Family Scombridae**						
*Auxis thazard*	2	0.1	2.0**^a^**	−	−	Iñiguez et al. (2009)**^c^**, Sardella & Luque (2016)**^g^,**Sardella (2017)**^g^**
*Euthynnus alletteratus*	46	17.4	2.62±2.06	0.45±1.29	1.0-7.0	Alves & Luque (2006)
*Katsuwonus pelamis*	15	40.0	2.67±2.73	1.06±2.12	1.0-8.0	Alves & Luque (2006)
*Scomberomorus cavalla*	30	1.0	2.0**^a^**	0.02	−	Dias et al. (2011)
*Scomber colias*	50	8.0	−	−	−	Rego & Santos (1983)
	100	4.0	3.5±3.3	0.1±0.8	1.0-8.0	Abdallah et al. (2002)
	100	4.0	3.5±3.3	0.1±0.8	1.0-8.0	Alves et al. (2003)
*Scomber scombrus*	43	25.6	2.38±2.10	0.72±1.57	1.0-7.0	Alves & Luque (2006)

**Order Siluriformes**						
**Family Doradidae**						
*Oxydoras niger*	20	5.0	−	−	−	Rodrigues et al. (2015)
**Family Pimelodidae**						
*Brachyplatystoma filamentosum*	22	9.09	−	−	−	Rodrigues et al. (2015)
*Brachyplatystoma rousseauxii*	40	15.0	12.0	1.8	3.0-21.0	Salgado (2011)
*Hypophthalmus marginatus*	11	85.71	21.0**^a^**	−	1.0-7.0	Cárdenas et al. (2021)
*Pimelodus blochii*	120	100	−	−	−	Cavalcante et al. (2020)

**Order Tetraodontiformes**						
**Family Balistidae**						
*Balistes vetula*	30	16.7	1.3±0.5	0.3±1.2	−	Alves et al. (2005)
**Family Monacanthidae**						
*Aluterus monoceros*	100	1.0	2.0**^a^**	0.02	−	Dias et al. (2010)
**Family Tetraodontidae**						
*Colomesus psittacus*	50	12.0	0.32	0.24	−	Giese et al. (2023)

**Order Zeiformes**						
**Family Zeidae**						
*Zenopsis conchifer*	10	1.0	1.0**^a^**	−	−	Sardella & Luque (2016)**^g^**, Sardella (2017)**^g^**

**Subclass Elasmobranchii**						
**Order Carcharhiniformes**						
**Family Carcharhinidae**						
*Carcharhinus signatus*	5	60.0	1.3	−	−	Knoff et al. (2001)
**Family Triakidae**						
*Galeorhinus galeus*	37	8.1	4.0	−	−	Knoff et al. (2001)
*Mustelus canis*	37	5.4	1.0	−	−	Knoff et al. (2001)

**Order Hexanchiformes**						
**Family Hexanchidae**						
*Heptranchias perlo*	7	14.3	1.0**^a^**	−	−	Knoff et al. (2001)
*Hexanchus griseus*	1	100	15.0**^a^**	−	−	Knoff et al. (2001)

**Order Rajiformes**						
**Family Rajidae**						
*Dipturus trachyderma*	8	25.0	1.0	−	−	Knoff et al. (2001)

**Order Squaliformes**						
**Family Squalidae**						
*Squalus megalops*	14	7.1	1.0**^a^**	−	−	Knoff et al. (2001)
*Squatina* sp.	20	3.8	1.0**^a^**	−	−	Knoff et al. (2001)

Abbreviations: *n*: Number of fish; P: Prevalence; MI: Mean intensity; MA: Mean abundance; SD: Standard deviation; RI: Range of infection.

a I: intensity;

b Identified as *Anisakis simplex*;

c Identified as *Anisakis typica*;

d Marajó Bay and Tapajós River respectively;

e Rio de Janeiro and Rio Grande do Sul respectively;

f Identified as *Anisakis* sp. larva type I sensu Berland (1961);

g Identified as *Skrjabinisakis physeteris*;

h Cabo Frio and Niterói respectively;

i Identified as *Anisakis typica*.

## Discussion

The third-stage larvae of *Anisakis* have an oval, transverse mouth opening surrounded by three poorly developed lips, an excretory pore at the cephalic end slightly below the larval tooth, a slender muscular esophagus, a present ventriculus, and a tail with or without a terminal mucron (Moravec, 1998; Timi et al., 2001; Felizardo et al., 2009; Fonseca et al., 2016).

We agree with Moravec (1998) and Moravec et al. (2016) in stating that the systematics of parasitic anisakids have been based on adult morphology; the systematics of the larvae, however, are underdeveloped, making it impossible to assign more specific taxonomic levels to the larval stages. Berland (1961) morphologically described two larval types of *Anisakis*: Larval morphotype I, characterized by an elongated ventriculus, an oblique ventriculus-intestinal junction, and a rounded tail with a mucron; larval morphotype II, with a short ventriculus, a horizontal junction between the ventriculus and intestine, an elongated, conical tail, and no mucron.

Murata et al. (2011), in their study of morphological and molecular characterization, described and identified at the species and/or species group level, four larval morphotypes of *Anisakis*: Morphotype I is characterized by a long ventriculus, oblique ventriculus-intestinal junction, and short and rounded tail with mucron, these morphological characteristics being attributed to the species *Anisakis simplex* s.s., *A*. *pegreffii*, *A*. *berlandi*, *A*. *typica*, *A*. *ziphidarum*, and *A*. *nascettii*; morphotypes II, III, and IV present a short ventriculus and junction between ventriculus and horizontal intestine, however, with distinct caudal morphologies. Morphotype II—long, conical, tapered tail without mucron (attributed to *S*. *physeteris*); morphotype III—short, rounded tail without mucron (attributed to *S*. *brevispiculata*), with 2 larvae presenting a tiny spine-like mucron, suggesting that it may be another species; and morphotype IV—short, conical, and pointed tail without mucron (*S*. *paggiae*) (see Murata et al., 2011). Chero et al. (2023) characterized three types of *Anisakis* larvae (morphotype I larvae with an elongated ventriculus, short, rounded tail, and conspicuous mucron; morphotype II with a short, oblong ventriculus, elongated tail, and no mucron; and morphotype III larvae with a short, oblong ventriculus, short, conical tail, and no mucron), which were molecularly identified as *Anisakis pegreffii*, *Skrjabinisakis physeteris*, and *S*. *brevispiculata*, respectively. In this work, we will accept the validity of *Skrjabinisakis* (*S*. *physeteris*, *S*. *brevispiculata,* and *S*. *paggiae*) (Safonova et al., 2021), but we will follow Takano & Sata (2022) and Chero et al. (2023) by not relocating *A*. *typica* to the genus *Peritrachelius* without there being strong molecular evidence for this assertion.

According to these characteristics and the records in the literature, the presence of three larval morphotypes of *Anisakis* in fish in Brazil is suggested: morphotypes I, II, and III. However, we highlight the need for further studies to characterize the occurrence of the larval stages (L3) of *Anisakis* spp., since there are records of adults of *S*. *paggiae* reported parasitizing cetaceans in Brazilian waters (Di Azevedo et al., 2015, 2017), which present larvae with morphological characteristics of morphotype IV (Takano et al., 2021, 2024), suggesting that this larval morphotype also occurs in Brazil but has not yet been reported. Morphological and morphometric data on third-stage larvae of *Anisakis* parasitizing fish in Brazil are shown in Table 2.

The diversity of fish infected by *Anisakis* larvae in Brazil (Table 1) is represented by 18 orders, 2 groups, 40 families, 60 genera, and 69 species. The larvae recovered from this ichthyofauna present great morphological and morphometric similarities among themselves, although they are described in different hosts and localities (Table 2). According to Mattiucci et al. (2018), members of the genus *Anisakis* have a global distribution and have been genetically confirmed in more than 40 species of final hosts and more than 160 species of intermediate/paratenic hosts. Recent research on the group suggests that species-level classification based solely on morphological and morphometric data, without proper molecular information, may not be appropriate. Molecular information is essential for identifying species and their hybrid forms (Moravec et al., 1993; Klimpel & Palm, 2011; Mostafa et al., 2020; Nonković et al., 2025), especially when mitochondrial and nuclear markers are used jointly (D’Amelio et al., 2000; Blažeković et al., 2015; Palomba et al., 2020; Hrabar et al., 2021).

The prevalence of *Anisakis* larval infection is presented in a diverse manner in parasitic literature, with the highest prevalences (<70%) recorded for *Lutjanus jocu*, *Lutjanus purpureus*, *Lutjanus vivanus*, and *Hypophthalmus marginatus*, with a prevalence of 100% for *Plagioscion squamosissimus*, *Pimelodus blochii*, and *Hexanchus griseus*. When we analyzed the occurrence and prevalence of *Anisakis* larvae in fish from the northern region of Brazil, only the states of Acre, Amazonas, and Pará recorded the presence of *Anisakis* larvae in commercially important fish (*Cichla monoculus*, *Plagioscion squamosissimus*, *Oxydoras niger*, *Brachyplatystoma filamentosum,* and *Brachyplatystoma rousseauxii*) (Table 3), with emphasis on the order Siluriformes and the families Ariidae, Doradidae, and Pimelodidae because they are composed of important species captured in the industrial fishing of large Amazon catfish. Pavanelli et al. (2013) state that less than 25% of the Brazilian ichthyofauna has been studied with the objective of understanding its parasitic fauna, with the Amazon region being one of the most important in generating research on parasites of aquatic organisms. However, other regions of Brazil remain a vast field to be explored.

The higher prevalence of parasitism in marine fish (62%) and the lower distribution pattern of third-stage *Anisakis* larvae in freshwater fish may be related to differences in the life history of the fish, the process of coadaptation and coevolution between host and parasite, and/or interspecific competition, which can reduce the range of potential hosts in sympatric conditions. Additionally, there is a lower diversity of definitive hosts in the biological cycles of *Anisakis* in this habitat. According to EFSA in 2010 (Allende et al., 2024), no marine fishery can be considered free of *A. simplex* larvae, and that all wild saltwater and freshwater fish should be considered at risk of containing viable larvae that pose a danger to human health if these products are consumed raw or almost raw.

*Trichiurus lepturus* and *Pagrus pagrus* were the species with the highest number of records in the parasitic literature for *Anisakis* larvae; *Scomber colias* presented the highest number of infection sites. Regarding their feeding habits, these three species are carnivorous/piscivorous. According to Froese & Pauly (2024), although the three species have a piscivorous feeding habit as adults, during the juvenile phase they are generalist feeders, preying on crustaceans (euphausiids and copepods), mollusks, fish, and occasionally squid. The presence of a vast parasitic record for the three species may be related to the position they occupy in the trophic chains in marine environments, since, according to Marcogliese (1995), the biological cycle of *Anisakis* is heteroxenous, with planktonic crustaceans acting as intermediate hosts, fish and cephalopods as paratenic hosts, and marine mammals such as dolphins, porpoises, and mainly toothed whales acting as definitive hosts.

The biological cycle of *Anisakis* spp. larvae begins with the release of eggs in the feces of cetaceans, where they are embryonated, after two moults, the eggs hatch into free-swimming third-stage larvae (L3) and release larvae that are ingested by planktonic crustaceans (especially euphausiids and less frequently mysids), migrating to the hemocoel, developing further, and becoming infective. When crustaceans are ingested by fish and/or cephalopods (paratenic hosts), the larvae migrate from the digestive tract to the visceral cavity and occasionally to the skeletal musculature, where they spiralize and remain in a state of paratenesis until the paratenic host is ingested by a final host (Nonković et al., 2025). When paratenic hosts are preyed upon by larger fish, digestive tract migration and spiralization into visceral organs occur again, resulting in the accumulation of many parasites along the food webs (Nagasawa, 1990; Marcogliese, 1995; Køie, 2001; Klimpel et al., 2004, 2011; Klimpel & Palm, 2011; Gregori et al., 2015). This may be the case with *Scomber colias*.

Worldwide, fish consumption has grown, and the expansion of the consumption of raw and/or lightly preserved fish and seafood (cold marinated, cold smoked, lightly salted) has influenced the increase in foodborne diseases, accounting for 420,000 deaths in 2010 and a global burden of 33 million disabled people (DALYs) (FAO, 2014). In this regard, Eiras (2024) highlights that the most important and fundamental characteristic of these infections is that they cannot occur unless there is some “cooperation” by humans, i.e., by ingesting raw or improperly cooked, preserved infected fish. Carvalho et al. (2020) described parasitism by *Anisakis* larvae in ducks raised on Marajó Island, which had access to fresh fish viscera, erratically entering the life cycle of these parasites. This raises an alert to the population regarding the need for sanitary care and proper disposal of fish viscera on the island.

The Amazon region is the largest producer of freshwater fish in Brazil (WWF, 2023), and its residents are among the largest consumers of protein from fishing worldwide (Batista & Petrere, 2003; Brabo et al., 2021), with per capita fish consumption rates ranging from 51 kg/year to 266 kg/year (Lopes, 2023). This exceeds the national per capita consumption of 11.17 kg/inhabitant/year (Lopes et al., 2016) and the world per capita consumption of 20.2 kg/inhabitant/year (FAO, 2022), reaffirming the importance of Amazonian fisheries resources, not only as food, but also from a socioeconomic, ecological, and cultural perspective (Dias et al., 2023).

According to the 1988 standardized nomenclature of parasitic zoonoses, anisakidosis (infection by Anisakidae worms) and anisakiasis (infection by *Anisakis* worms) is an underdiagnosed, emerging, and cosmopolitan ichthyozoonosis resulting from the accidental ingestion of the third larval stage (L3) of parasitic nematodes belonging to the family Anisakidae (*Anisakis*, *Pseudoterranova,* and *Contracaecum*), with anisakiasis caused by *Anisakis* spp. being the predominant form of the disease, originated by the consumption of fish and/or cephalopods infected with these larvae (Eiras et al., 2015; Adroher et al., 2024; Nonković et al., 2025).

In Brazil, despite high fish consumption, the Ministry of Health, in 2022, classified the biological risk of Anisakidae infection as belonging to Risk Class 2, since these parasites are considered to pose a moderate individual risk and a limited risk of transmission (Brasil, 2022). Infections by *Anisakis* larvae in humans result from a combination of factors: direct action of the larvae during tissue invasion and interactions between the host's immune system and the substances released by the parasite or the host's immune response to its presence (Martínez Ubeira & Valiñas Sobral, 2000). Asymptomatic infections can occur when the larvae remain within the gastrointestinal lumen without any adverse impact on the host's health (Cong & Elsheikha, 2021).

The first record of Anisakidae parasitizing humans was documented in 1876 by Leuckhart (Hoyle & Leuckhart, 1886). It was mentioned again in 1960, after several people consumed salted herring in the Netherlands, Van Thiel noted and described the “very unusual finding” of a marine worm (herring fluke) in the center of an eosinophilic granuloma in a patient with acute abdominal pain (Van Thiel, 1960); later, this nematode was identified as *Anisakis* sp. larvae, with the majority of human infections being associated with *Anisakis simplex*.

Anisakiasis is a little-recorded disease in Brazil (Figueiredo et al., 2013a; Santos et al., 2020), although the case of nine people who consumed raw fish of the genus Cichlydae on Bananal Island, Tocantins state after a fishing trip and after approximately 20 days, five of these people became ill, this interval corresponds to the time for clinical manifestation in infected people and for possible allergic manifestations, however the larvae were not recovered in the digestive tract, the clinical evidence of three patients and the hematological alterations suggested a diagnosis of anisakiasis (Amato et al., 2007). Cruz et al. (2010) reported the presence of a 1.5 cm larva with a filiform appearance, which was observed in an endoscopic examination of the duodenum causing inflammation of the mucosa, in a 73-year-old man who traveled through the state of Bahia and ingested seafood, later showing clinical signs of the disease, thus confirming the first record of *Anisakis* sp. in Brazil, with the observation of the larva.

The consumption of fish without due hygienic and sanitary care in Brazil hinders food safety, and there is an urgent need for this to be understood at all levels of academia, government, industry, politics, and research as a public health policy due to the inseparable relationship of the parasite-host-environment triad (Boqvist et al., 2018). The Anisakidae nematodes pose a harmful threat to populations that consume fish and represent a biological risk associated with the consumption of these aquatic resources (Polimeno et al., 2021).

The presence of anisakid larvae (L3) is extremely important, as they cause lesions in fish tissue that lead to fish mortality, resulting in enormous economic losses for the fishing industry (Cárdenas et al., 2021). In addition, they are important pathogens that cause foodborne diseases (Chero et al., 2023). The occurrence of anisakiasis can be prevented by abstaining from eating raw or undercooked fish, cooking at 70°C for at least one minute (Alves & Santos, 2016), or by immediately eviscerating the fish after capture to prevent the migration of *Anisakis* larvae from the viscera to the muscles (Smith & Wootten, 1975; Acha & Szyfres, 2003).

In the industry, fish must be visually inspected, and infected fish must not be marketed (Hartmann & Matern, 1988; European Union, 2004). These fish can be subjected to different processes such as freezing at –20 °C for 72 hours, candling, hydraulic pressing (most commonly used), inspection under ultraviolet light, and digestion, both recommended by the Codex (ISO 23036-1:2021, Part I and II); spectral imaging; electromagnetic detection of parasites; and molecular analysis (Karl & Leinemann, 1993; Choudhury et al., 2002; Heia et al., 2007; Sivertsen et al., 2012; Llarena-Reino et al., 2013; Šimat et al., 2015; Cammilleri et al., 2016; Gómez-Morales et al., 2018; Chalmers et al., 2020). Additionally, even when the parasite is detected, there is still a risk of allergens remaining in the food, and some allergens are heat-resistant; for example, pepsin from *A*. *simplex* (Anis 4) has already been recorded in commercial flour used in the production of fish and chicken feed, which confirms the transfer of the allergen to fishmeal (Polimeno et al., 2021).

## Conclusions

In this paper, we present the morphological, morphometric, biogeographic, and prevalence data of *Anisakis* larvae, highlighting their zoonotic potential in marine, brackish, and freshwater fish in Brazil. We emphasize the importance of the fish that make up the Amazonian ichthyofauna, not only due to their high consumption in the region but also because they are integral to the local economy, resource generation, and the cultural heritage of traditional and riverside populations. To ensure safe fish consumption, aquaculture can serve as a safe food source by ensuring that the cultivated resources are free of these zoonotic parasites.

## Data Availability

Data will be made available on request.
